# Retraction: Machine learning-based anomaly detection and prediction in commercial aircraft using autonomous surveillance data

**DOI:** 10.1371/journal.pone.0324279

**Published:** 2025-05-09

**Authors:** The PLOS One Editors

The *PLOS One* Editors retract this article [1] due to concerns about authorship and potential manipulation of the publication process. We regret that the issues were not identified prior to the article’s publication.

All authors either did not respond directly or could not be reached.
